# Donor and Recipient Polygenic Risk Scores Influence Kidney Transplant Function

**DOI:** 10.3389/ti.2025.14171

**Published:** 2025-03-04

**Authors:** Kane E. Collins, Edmund Gilbert, Vincent Mauduit, Katherine A. Benson, Elhussein A. E. Elhassan, Conall O’Seaghdha, Claire Hill, Amy Jayne McKnight, Alexander P. Maxwell, Peter J. van der Most, Martin H. de Borst, Weihua Guan, Pamala A. Jacobson, Ajay K. Israni, Brendan J. Keating, Graham M. Lord, Salla Markkinen, Ilkka Helanterä, Kati Hyvärinen, Jukka Partanen, Stephen F. Madden, Matthew B. Lanktree, Sophie Limou, Gianpiero L. Cavalleri, Peter J. Conlon

**Affiliations:** ^1^ School of Pharmacy and Biomolecular Sciences, Royal College of Surgeons in Ireland, Dublin, Ireland; ^2^ The Science Foundation Ireland FutureNeuro Centre of Excellence, Dublin, Ireland; ^3^ SFI Centre for Research Training in Genomics Data Science, University of Galway, Galway, Ireland; ^4^ Ecole Centrale Nantes, INSERM, Center for Research in Transplantation and Translational Immunology, UMR1064, Nantes University, Nantes, France; ^5^ Department of Nephrology and Transplantation, Beaumont Hospital, Dublin, Ireland; ^6^ Department of Medicine, Royal College of Surgeons in Ireland, Dublin, Ireland; ^7^ Centre for Public Health, Queen’s University Belfast, Belfast, United Kingdom; ^8^ Department of Epidemiology, University Medical Center Groningen, University of Groningen, Groningen, Netherlands; ^9^ Department of Internal Medicine, Division of Nephrology, University Medical Center Groningen, University of Groningen, Groningen, Netherlands; ^10^ Division of Biostatistics and Health Data Science, School of Public Health, University of Minnesota, Minneapolis, MN, United States; ^11^ College of Pharmacy, University of Minnesota, Minneapolis, MN, United States; ^12^ Internal Medicine, University of Texas Medical Branch, Galveston, TX, United States; ^13^ Department of Surgery, Perelman School of Medicine, University of Pennsylvania, Philadelphia, PA, United States; ^14^ School of Immunology and Microbial Sciences, King’s College London, London, United Kingdom; ^15^ Finnish Red Cross Blood Service, Research and Development, Biomedicum 1, Helsinki, Finland; ^16^ Helsinki University Hospital, Transplantation and Liver Surgery, Helsinki, Finland; ^17^ Data Science Centre, Beaux Lane House, Royal College of Surgeons in Ireland, Dublin, Ireland; ^18^ Division of Nephrology, Departments of Medicine and Health Research Methodology, Evidence and Impact, St. Joseph’s Healthcare Hamilton, McMaster University and Population Health Research Institute, Hamilton, ON, Canada

**Keywords:** polygenic risk scores, eGFR, graft survival, graft function, multivariable models

## Abstract

Kidney transplant outcomes are influenced by donor and recipient age, sex, HLA mismatch, donor type, anti-rejection medication adherence and disease recurrence, but variability in transplant outcomes remains unexplained. We hypothesise that donor and recipient polygenic burden for traits related to kidney function may also influence graft function. We assembled a cohort of 6,060 living and deceased kidney donor-recipient pairs. We calculated polygenic risk scores (PRSs) for kidney function-related traits in both donors and recipients. We investigated the association between these PRSs and recipient eGFR at 1- and 5-year post-transplant as well as graft failure. Donor: hypertension PRS (*P* < 0.001), eGFR PRS (*P* < 0.001), and intracranial aneurysm PRS (*P* = 0.01), along with *recipient* eGFR PRS (*P* = 0.001) were associated with eGFR at 1-year post-transplantation. Clinical factors explained 25% of the variation in eGFR at 1-year and 13% at 5-year, with PRSs cumulatively adding 1% in both cases. PRSs were not associated with long-term graft survival. We demonstrate a small, but statistically significant association between donor and recipient PRSs and recipient graft function at 1- and 5-year post-transplant. This effect is, at present, unlikely to have clinical application and further research is required to improve PRS performance.

## Graphical Abstract

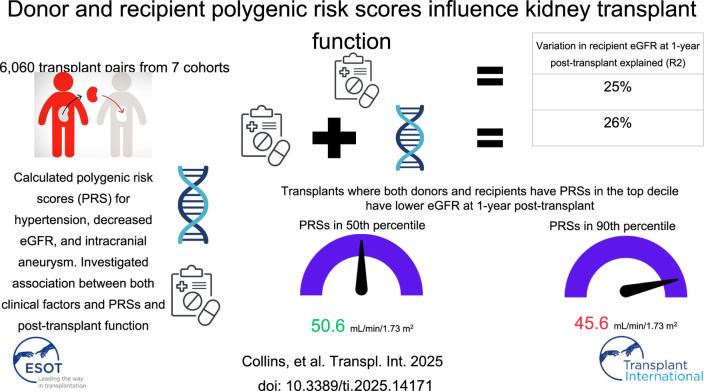

## Introduction

Kidney transplant outcomes are influenced by a wide array of factors including donor age and sex, whether the donor is living or deceased, clinical era of transplant, donor cause of death, and HLA mismatch [1, 2]. While significant progress has been made in improving short-term graft survival, enhancing medium- and long-term graft survival and function still remains a challenge [3].

HLA mismatch and blood group are the only genetic factors currently used in transplant allocation decisions. It is well established that graft survival is inversely related to the number of mismatched HLA alleles [4]. However, in many centres, less than 5% of transplants are fully matched across the 6 HLA antigens tested [5]. Thanks to modern immunosuppression, it is possible to have good outcomes even with poorly HLA matched kidneys [6]. It has also been reported that mismatches between donor and recipient in non-synonymous single nucleotide polymorphisms (SNPs) in genes for transmembrane and secreted proteins and outside the HLA were significantly associated with graft survival [7]. However, a subsequent replication attempt, involving nearly 8,000 pairs, found no significant associations between these variants and graft outcome [8]. A more recent study reported an association between donor and recipient genetic mismatch and graft survival [9]. Genetic mismatch in this context was defined as the sum of variant mismatches in transmembrane, secretory, and kidney-related proteins.

Polygenic risk scores (PRSs) quantify individual genetic burden for a trait using summary statistics from genome-wide association studies (GWAS). Specifically, they estimate the cumulative effect of common genetic variation on an individual’s disease status weighted by estimated effect size [10].

PRSs for various traits of the donor kidney (“donor PRS”) have been reported to be associated with transplant outcome. Donor burden for estimated glomerular filtration rate (eGFR) has been correlated with eGFR post-transplant [11]. Other studies have shown an association between donor genetic risk scores in interleukin-6 and biopsy proven rejection [12, 13]. A recent study from our group has shown that donor kidneys in the top decile of PRS for traits related to stroke have eGFR at 1-year post-transplant approximately 5 mL/min/1.73 m^2^ lower than those in the bottom decile of risk [14]. The effect of recipient polygenic burden on transplant outcome has also been established for several outcomes of interest. Recipient polygenic burden of eGFR has been shown to be associated with post-transplant eGFR [11] and recipient burden for skin cancer has been associated with skin cancer post-transplant [11, 15]. Recipient PRS for type 2 diabetes was shown to be associated with the development of post-transplant diabetes, and the same study found that the same PRS in donors was a significant predictor of post-transplant diabetes, but only in liver transplants [16]. Shaked et al also found that combining both donor and recipient PRS for type 2 diabetes significantly improved type 2 diabetes prediction [16].

We assembled 6,060 genotyped donor-recipient transplant pairs across seven cohorts. We calculated kidney function related PRS for seven traits in both donors and recipients. We test the correlation between polygenic burden and transplant outcomes, particularly eGFR at 1 and 5 years post-transplant, as well as long-term graft survival.

## Materials and Methods

### Inclusion Criteria

The inclusion criteria were as follows: (1) availability of SNP array genotyping data for both the donor and recipient in a transplant pair; (2) availability of data on donor age, sex, and kidney donation type (living, deceased of stroke, deceased of other cause), recipient age, sex, year of transplant, and whether it was the recipient’s first transplant; (3) at least one of the following outcome variables was also required: death-censored graft survival, eGFR at 1-year post-transplant, eGFR at 5-year post-transplant (plus or minus 3 months for each). If a graft had failed by 1- or 5-year, then individuals were assigned values of eGFR at 1- or 5-year respectively of 0 mL/min/1.73 m^2^.

### Patient Cohort Descriptions

We included seven predominantly European ancestry cohorts from the following regions: USA: Deterioration of Kidney Allograft Function (DeKAF), Genomics of kidney transplantation (GEN03). Finland: Finnish Red Cross Blood Service (FRCBS). Netherlands: Transplant Lines (TL). France: Kidney Transplantation - Genomic Investigation of Essential Clinical concerns (KiT-GENIE). UK and Ireland: United Kingdom and Ireland Renal Transplant consortium (UKIRTC), Queen’s University Belfast (QUB). See Supplementary Materials for more detailed information on the recruitment and characteristics of each of these cohorts involved. There were 924 missing values of HLA mismatch, so we performed multiple imputation using the R package mice based on the variables for donor type, donor age, recipient age, donor sex, recipient sex, and whether it was the recipient’s first transplant.

### Calculation of PRS

SNP array genotype data was subject to quality control for minor allele frequency, missingness per marker, and missingness per individual (see Supplementary Materials). We calculated PRSs in each individual for hypertension [17], eGFR [18], rapid decline in eGFR [19], albuminuria [20], total kidney volume (TKV) [21], stroke [22], and intracranial aneurysm [23] using published GWAS for each trait. These traits were selected as they were directly related to kidney function and risk factors for progression of kidney disease. We have previously demonstrated the impact of donor intracranial aneurysm and stroke as a cause of death to be associated with recipient graft function [14]. Further details of these GWAS can be found in Supplementary Table S1. PRSs were calculated using PRSice2 [24], selecting alleles with a p-value threshold greater than 0.5 (see Supplementary Materials for further details). All analysis was conducted in R, using version 4.2.1 (2022-06-23) [25].

For two of these PRSs (eGFR and total kidney volume), we hypothesised that higher values would be associated with better kidney function [26], while for the others (hypertension, albuminuria, stroke, intracranial aneurysm, and rapid kidney function decline), one might expect that higher values would be associated with worse kidney function. To simplify interpretation, we standardised the directionality of all PRSs, such that one might expect higher scores to be associated with negative outcomes. We did this by inverting the sign of the eGFR and total kidney volume PRSs to create “new” PRSs, which we will refer to as “decreased eGFR,” and “decreased total kidney volume.”

### Univariable Analysis

A series of univariable linear models for recipient eGFR at 1- and 5-year post-transplant were created for all the clinical factors (donor age, donor sex, recipient age, recipient sex, HLA mismatch, year of transplant, donor type, and whether it was the recipient’s first transplant), as well as the donor PRSs, and recipient PRSs. The variance in the outcome explained (R^2^) was also calculated for each factor. In a similar manner, a series of univariable Cox proportional hazards models for death-censored graft survival were created for each of the clinical factors, donor PRSs, and recipient PRSs.

### Multivariable Analysis

For each of the three outcomes of interest in the univariable analysis (eGFR at 1-year, eGFR at 5-year, and graft survival), multivariable models were created with just the factors that had a p-value less than 0.2in the univariable analysis. Assumptions of a linear model (residuals vs. fitted, normal Q-Q, scale-location, and residuals vs. leverage) and Cox model (proportional hazards, nonlinearity and influential observations respectively) were also checked. The adjusted R^2^ for each model was also calculated. The adjusted R^2^ for a model without the PRSs (with just the clinical factors), was then calculated. Using the R function *anova*, an ANOVA test was then carried out to investigate if there was a statistically significant difference between these models.

### Comparison of Outcomes Between Individuals With High and Low Polygenic Burden

We used these multivariable linear models to predict eGFR at 1- and 5-year for two transplant recipients: one with high PRSs (in the 90th percentile), and the other with average PRSs (in the 50th percentile), but are otherwise completely identical. We did this for the median transplant recipient, which in our cohort, took place in 2007, with a 51 year old male recipient, on his first transplant, with a 50 year old male donor who died of stroke, with whom he has three HLA mismatches.

## Results

Table 1 shows the characteristics for the 6,060 kidney transplants ascertained from seven sites that passed genotyping quality control. The median donor age was 50 years and there were more males (3,221, 53%) than females. 1,470 (24%) of the donors were living, 2,826 (47%) died of stroke, while 1,764 (29%) died of other causes. The median recipient age was 51 years, with more male recipients than female (3,912, 65%). First transplants comprised 88% of the cohort, and HLA mismatch data was available for 85% of the cohort. The median number of HLA mismatches in a donor: recipient pair was 3. One-year graft survival was 97%, 5-year graft survival was 89%, and 10-year graft survival was 76%. Recipient eGFR at 1-year post-transplant was available for 88% of the cohort, with a median of 52 mL/min/1.73 m^2^ while eGFR at 5-year post-transplant was only available for 50% of the cohort, with a median of 44 mL/min/1.73 m^2^. Power calculations indicated that the smallest sample size required to detect an effect that explains at least 1% of the variation in outcome was 272 individuals (see Supplementary Materials).

**TABLE 1:** Demographic characteristics of study participants. For further details regarding the recruitment and characteristics of each cohort, see the Supplementary Materials.

Variable	Overall	DeKAF	FRCBS	GEN03	KiT-GENIE	QUB	TL	UKIRTC
Number of transplants	6,060	684	888	472	1,463	68	608	1877
Donor age, median (range)	50 (18–90)	44 (18–70)	58 (18–77)	45 (18–71)	56 (18–90)	44 (18–66)	46 (18–72)	47 (18–81)
Female donor, n (%)	2,839 (47)	405 (59)	417 (47)	271 (57)	616 (42)	32 (47)	292 (48)	806 (43)
Donor type								
*Living, n (%)*	1,470 (24)	684 (100)	0 (0)	472 (100)	265 (18)	0 (0)	49 (8)	0 (0)
*Died of stroke, n (%)*	2,826 (47)	0 (0)	585 (66)	0 (0)	699 (48)	42 (62)	319 (52)	1,181 (63)
*Died of other causes, (n %)*	1764 (29)	0 (0)	303 (34)	0 (0)	499 (34)	26 (38)	240 (39)	696 (37)
Recipient age, median (range)	51 (0–84)	51 (0–83)	57 (18–79)	51 (1–81)	55 (18–84)	44 (10–72)	50 (16–74)	47 (18–79)
Female recipient, n (%)	2,148 (35)	231 (34)	275 (31)	177 (38)	497 (34)	30 (44)	246 (40)	692 (37)
First transplant, n (%)	5,337 (88)	603 (88)	888 (100)	418 (89)	1,140 (78)	68 (100)	555 (91)	1,665 (89)
HLA mismatch, median (range)	3 (0–6)	3 (0–6)	3 (0–6)	3 (0–6)	4 (0–6)	NA	NA	2 (0–6)
*Unknown*	924 (15)	1 (0.1)	0 (0)	13 (3)	0 (100)	68 (100)	608 (100)	234 (12)
Year of transplant, median	2007	2008	2014	2014	2011	2002	2000	2001
Follow up, median (range)	5 (0–25)	2 (0–5)	3 (0–10)	2 (0–3)	6 (0–21)	7 (0–24)	7 (0–17)	8 (0–25)
Graft status, n (%)								
*Censored*	5,098 (84)	671 (98)	831 (94)	470 (100)	1,138 (78)	46 (68)	509 (84)	1,433 (76)
*Rejected*	962 (16)	13 (2)	57 (6)	2 (0.4)	325 (22)	22 (32)	99 (16)	444 (24)
eGFR at 1-year, median (range)	52 (0–185)	60 (0–178)	54 (0–135)	62 (16–185)	50 (0–129)	0 (0–0)	45 (0–124)	49 (0–124)
*Unknown, n (%)*	726 (12)	0 (0)	247 (28)	0 (0)	59 (4)	53 (78)	24 (4)	343 (18)
eGFR at 5-year, median (range)	44 (0–124)	0 (0–0)	38 (0–106)	0 (0–0)	45 (0–122)	0 (0–0)	47 (0–124)	44 (0–121)
*Unknown, n (%)*	3037 (50)	671 (98)	747 (84)	470 (99.5)	491 (34)	49 (72)	139 (23)	470 (25)

DeKAF, deterioration of kidney allograft function; FRCBS, finnish red cross blood service; GEN03, genomics of kidney transplantation; KiT-GENIE, kidney transplantation - genomic investigation of essential clinical concerns; QUB, Queen’s University Belfast; TL, TransplantLines; UKIRTC, united kingdom and ireland renal transplant consortium; eGFR, estimated glomerular filtration rate.

### Univariable Models to Identify Factors Associated With Transplant Outcome

In order to investigate the impact of donor and recipient PRSs on eGFR at 1- and 5-year, we created univariable linear models for each PRS. Similarly, we also created univariable Cox models for each PRS to predict graft failure (see *Materials and Methods*). The association between each of the clinical factors, seven donor PRSs, and seven recipient PRSs and recipient eGFR at 1-year, 5-year, and graft failure are detailed in Table 2. We observed a significant univariable association between the following ***donor*** characteristics and recipient eGFR at 1-year: age (Estimate = −0.63; *P* < 2e-16), male sex (Estimate = 3.4; *P* = 6.1e-8), stroke cause of death (Estimate = −17; *P* < 2e-16), other cause of death (Estimate = −8.6; *P* < 2e-16), and year of transplant (Estimate = 0.45; *P* < 2e-16). Standard deviation increases in donor hypertension, decreased eGFR, and intracranial aneurysm PRSs correspond to decreases in eGFR at 1-year of 1.6 (*P* = 6.7e-7), 1.5 (*P* = 5.4e-6), and 1.0 (*P* = 0.001) mL/min/1.73 m^2^ respectively. We also observed significant associations between ***recipient*** age (Estimate = −0.46; *P* = 8.2e-12), recipient decreased eGFR PRS (Estimate = −1.5; *P* = 4.4e-4) and recipient eGFR at 1-year. None of the other PRSs were significantly associated with eGFR at 1-year. The factors with the highest R^2^ were donor age, recipient age, and donor type (0.16, 0.08, 0.10 respectively).

**TABLE 2:** Univariable linear models for recipient eGFR at 1- and 5-year post-transplant, and Cox model for death-censored graft failure.

	eGFR at 1-year			eGFR at 5-year			Graft failure	
	Estimate (SE)	*P* value	R^2^	Estimate (SE)	*P* value	R^2^	HR (95% CI)	*P* value
Clinical factors								
Donor age	***−0.63 (0.02)***	***<2e-16***	***0.16***	***−0.64 (0.03)***	***<2e-16***	***0.12***	***1.02 (1.02–1.03)***	***<2e-16***
Male donor sex	***3.4 (0.62)***	***6.1e-8***	***0.005***	***3.3 (0.98)***	***0.001***	***0.004***	0.98 (0.86–1.1)	0.72
Male recipient sex	1.2 (0.65)	0.06	0.001	***2.5 (1.01)***	***0.01***	***0.002***	1.1 (0.93–1.2)	0.34
Recipient age	***−0.46 (0.02)***	***<2e-16***	***0.08***	***−0.24 (0.04)***	***8.2e-12***	***0.02***	1.00 (0.99–1.01)	0.29
HLA mismatch	−0.16 (0.20)	0.43	0	***−0.77 (0.33)***	***0.02***	***0.001***	***1.1 (1.02–1.11)***	*0.006*
First transplant	−0.2 (0.94)	0.83	0	***3.0 (1.38)***	***0.03***	***0.002***	***0.66 (0.56–0.78)***	***7.9e-7***
Year of transplant	***0.45 (0.047)***	***<2e-16***	***0.02***	−0.15 (0.08)	0.06	0.001	***0.98 (0.98–0.99)***	***0.003***
Donor type			***0.1***			***0.03***		
***Living***	***-***	***-***		***-***	***-***		*-*	*-*
*Stroke cause of death*	***−17 (0.72)***	***<2e-16***		***−13 (1.95)***	***1.7e-10***		***3.5 (2.5–4.7)***	***1.6e-14***
*Other cause of death*	***−8.6 (0.79)***	***<2e-16***		***−4.2 (2.01)***	***0.04***		***2.7 (2.0–3.8)***	***1.4e-9***
Donor PRSs								
Donor hypertension PRS	***−1.6 (0.32)***	***6.7e-7***	***0.005***	***−1.2 (0.49)***	***0.02***	***0.002***	***1.07 (1.00–1.14)***	***0.049***
Donor decreased eGFR PRS	***−1.5 (0.32)***	***5.4e-6***	***0.004***	***−1.8 (0.5)***	***4.4e-4***	***0.004***	1.05 (0.99–1.13)	0.11
Donor albuminuria PRS	0.52 (0.32)	0.1	0.001	−0.34 (0.5)	0.5	0	1.00 (0.94–1.06)	0.92
Donor rapid eGFR decline PRS	−0.37 (0.38)	0.34	0	−0.15 (0.51)	0.77	0	0.99 (0.92–1.06)	0.73
Donor intracranial aneurysm PRS	***−1.03 (0.31)***	***0.001***	***0.002***	−0.6 (0.48)	0.21	0.001	1.04 (0.98–1.11)	0.18
Donor stroke PRS	−0.12 (0.31)	0.7	0	0.58 (0.5)	0.23	0	0.95 (0.89–1.01)	0.09
Donor decreased TKV PRS	0.25 (0.31)	0.43	0	0.08 (0.5)	0.87	0	0.98 (0.92–1.05)	0.58
Recipient PRSs								
Recipient hypertension PRS	0.53 (0.30)	0.08	0	0.63 (0.49)	0.2	0.001	0.99 (0.92–1.1)	0.64
Recipient decreased eGFR PRS	***−1.5 (0.31)***	***1.0e-6***	***0.004***	***−1.1 (0.47)***	***0.02***	***0.002***	1.05 (0.99–1.1)	0.09
Recipient albuminuria PRS	0.56 (0.32)	0.08	0.001	0.14 (0.48)	0.77	0	1.02 (0.96–1.1)	0.5
Recipient rapid eGFR decline PRS	0.34 (0.36)	0.35	0	0.65 (0.48)	0.17	0.001	0.97 (0.91–1.0)	0.4
Recipient intracranial aneurysm PRS	0.12 (0.32)	0.7	0	−0.43 (0.5)	0.38	0	1.00 (0.94–1.1)	0.97
Recipient stroke PRS	−0.29 (0.32)	0.36	0	−0.83 (0.49)	0.09	0.001	1.10 (0.99–1.1)	0.12
Recipient decreased TKV PRS	0.1 (0.31)	0.74	0	0.1 (0.49)	0.83	0	0.94 (0.88–1.0)	0.06

eGFR, estimated glomerular filtration rate; HR, hazard ratio; TKV, total kidney volume; SE, standard error.

Statistically significant (*P* < 0.05) predictors are bolded and italicised.

Univariable ***donor*** factors associated with eGFR at 5-year post-transplant included: age (Estimate = −0.64; *P* < 2e-16), male sex (Estimate = 3.3; *P* = 0.001), stroke cause of death (Estimate = −13; *P* = 1.7e-10), other cause of death (Estimate = −4.2; *P* = 0.04), and HLA mismatch (Estimate = −1.1; *P* = 0.002). Standard deviation increases in donor hypertension PRS, donor decreased eGFR PRS, and recipient decreased eGFR PRS correspond to decreases in eGFR at 5-year of 1.2 (*P* = 0.02), 1.8 (*P* = 4.4e-4), and 1.1 (*P* = 0.02) mL/min/1.73 m^2^ respectively. ***Recipient*** factors included whether it was the recipient’s first transplant (HR = 0.66; *P* = 7.9e-7), age (Estimate = −0.24; *P* = 8.2e-12), and male sex (Estimate = 2.5; *P* = 0.01).

Univariable ***donor*** factors associated with graft failure included age (HR = 1.02; *P* < 2e-16), HLA mismatch (HR = 1.1; *P* = 8.1e-5), stroke cause of death (HR = 3.5; *P* = 1.6e-14), other cause of death (HR = 2.7; *P* = 1.4e-9), year of transplant (HR = 0.98; *P* = 0.003), and hypertension PRS (HR = 1.07; *P* = 0.049). No recipient factors were associated with graft failure. A standard deviation increase in donor hypertension PRS corresponds to a 7% greater risk of graft failure.

### Multivariable Models to Identify Factors Associated With Transplant Outcome

For each of the three outcomes of interest (eGFR at 1-year, 5-year and graft failure), multivariable models were created using only the statistically significant factors from the univariable analysis (Table 3).

**TABLE 3:** Multivariable models for recipient eGFR at 1- and 5-year post-transplant, and graft failure, keeping statistically significant factors from univariate models. Effect of polygenic risk scores is highlighted in grey.

eGFR at 1-year (adjusted R^2^ = 0.26)		
	Estimate (95% CI)	*P* Value
Intercept	−1,267 (−1,460, −1,098)	<2e-16
Donor age	−0.54 (−0.59, −0.50)	<2e-16
Male donor sex	2.7 (1.5, 3.7)	21.6e-6
Male recipient sex	1.68 (0.52, 2.72)	0.004
Recipient age	−0.25 (−0.29, −0.21)	<2e-16
Year of transplant	0.68 (0.59, 0.78)	<2e-16
Donor type		
*Living*	-	-
*Stroke cause of death*	−7.6 (−9.1, −6.2)	<2e-16
*Other cause of death*	−6.3 (−7.8, −4.7)	<2e-16
Donor albuminuria PRS	0.24 (−0.32, 0.80)	0.40
Donor hypertension PRS	−1.3 (−1.7, −0.6)	9.2e-6
Donor decreased eGFR PRS	−1.2 (−1.8, −0.7)	4.33e-5
Donor intracranial aneurysm PRS	−0.66 (−1.2, −0.14)	0.01
Recipient hypertension PRS	0.47 (−0.07, 1.02)	0.09
Recipient albuminuria PRS	−0.05 (−0.62, 0.50)	0.85
Recipient decreased eGFR PRS	−1.0 (−1.5, −0.49)	0.001

eGFR at 5-year (Adjusted R^2^ = 0.14)		
	Estimate (95% CI)	*P* value
Intercept	−621 (−952, −290)	0.0002
Donor age	−0.68 (−0.7, −0.6)	<2e-16
Male donor sex	1.01 (−0.8, 2.9)	0.28
Recipient age	0.04 (−0.0, 0.1)	0.26
Male recipient sex	2.8 (0.9, 4.6)	0.004
Year of transplant	0.35 (0.2, 0.5)	3.7e-5
HLA mismatch	−0.61 (−1.3, 0.1)	0.09
First transplant	3.24 (0.7, 5.8)	0.01
Donor type		
*Living*	-	-
*Stroke cause of death*	−8.0 (−11.8, −2.8)	3.0e-5
*Other cause of death*	−6.7 (−10.5, −2.8)	0.0007
Donor hypertension PRS	−0.7 (−1.9, −0.1)	0.16
Donor decreased eGFR PRS	−1.6 (−2.6, −0.8)	0.0003
Recipient decreased eGFR PRS	−0.9 (−1.8, −0.0)	0.04
Recipient stroke PRS	−0.95 (−1.9, −0.1)	0.04

Graft failure(R^2^ = 0.23)		
	HR (95% CI)	*P* value
Donor age	1.02 (1.02, 1.03)	1.8e-13
HLA mismatch	1.13 (1.07, 1.19)	3.3e-6
First transplant	0.61 (0.52, 0.72)	6.1e-9
Year of transplant	0.97 (0.96, 0.98)	2.3e-8
Donor type		
*Living*	-	-
*Stroke cause of death*	2.6 (1.9, 3.6)	8.7e-9
*Other cause of death*	2.5 (1.8, 3.5)	5.6e-8
Donor Hypertension PRS	1.06 (0.99, 1.1)	0.09
Donor decreased eGFR PRS	1.04 (0.98, 1.1)	0.18
Donor intracranial aneurysm PRS	1.03 (0.96, 1.1)	0.39
Donor stroke PRS	0.95 (0.90, 1.01)	0.11
Recipient decreased eGFR PRS	1.05 (0.98, 1.11)	0.15
Recipient stroke PRS	1.06 (0.98, 1.12)	0.08
Recipient decreased TKV PRS	0.96 (0.90, 1.02)	0.17

eGFR, estimated glomerular filtration rate; CI, confidence interval; HR, hazard ratio.

In a multivariable model the following ***donor*** factors were independently associated with eGFR at 1-year: age, sex, year of transplant, donor type, hypertension PRS, decreased eGFR PRS, and intracranial aneurysm PRS. ***Recipient*** factors associated with eGFR at 1-year in the multivariable model included age, and decreased eGFR PRS. This model had an adjusted R^2^ of 0.26, compared to the adjusted R^2^ of a model with just the clinical factors of 0.25. There was a significant difference between the two models, according to the ANOVA test (F = 14.4, *P* = 9.9e-12), indicating that the addition of PRSs increases the predictive power of a model with just clinical factors.

In the multivariable model for eGFR at 5-year, ***donor*** factors associated included age, donor type, and decreased eGFR PRS. ***Recipient*** factors included sex, age, and decreased eGFR PRS. The adjusted R^2^ of the model with the PRSs was higher (0.14) than that of the model with just the clinical predictors (0.13). There was a significant difference between the two models, according to the ANOVA test (*P* = 0.003), again indicating that the addition of PRSs to a model of clinical factors significantly increases predictive ability.

The following factors were associated with graft failure in the multivariable model: donor age, HLA mismatch, whether it was the recipient’s first transplant, year of transplant, and donor cause of death. None of the PRSs were significantly associated with graft failure.

### Comparison of Outcomes Between Individuals With High and Low Polygenic Burden

To demonstrate the utility of these models, we used the models created in the previous section to predict recipient eGFR at 1- and 5-year post-transplant in the median transplant recipient (see *Materials and Methods*), one with high PRSs (in the 90th percentile) and the other with average PRSs (in the 50th percentile). Transplants where both the donor and recipient had high PRSs were predicted to have an eGFR at 1-year of 45.6 mL/min/1.73 m^2^, whereas transplants where both the donor and recipient had average PRSs were predicted to have an eGFR at 1-year of 50.6 mL/min/1.73 m^2^. Transplants where both the donor and recipient had high PRSs were predicted to have an eGFR at 5-year of 40.0 mL/min/1.73 m^2^, whereas transplants where both the donor and recipient had average PRSs were predicted to have an eGFR at 5-year of 42.8 mL/min/1.73 m^2^.

## Discussion

We have explored the influence of donor and recipient PRSs for traits related to kidney function on post-transplant outcome. We have confirmed the previously reported clinical factors associated with graft function, and have additionally demonstrated, across seven cohorts comprising 6,060 donor-recipient transplant pairs, that higher donor and recipient decreased eGFR PRS was associated with lower eGFR at 1-year post-transplant, with similar effects observed at 5-year post-transplant. We further demonstrated that donor hypertension and intracranial aneurysm PRSs are also associated with reduced eGFR at 1-year post-transplant. Transplants where both the donor and recipient had high polygenic burden were predicted to have recipient eGFR at 1-year post-transplant that was over 5 mL/min/1.73 m^2^ lower than those with average polygenic burden.

To our knowledge, this is the first study to combine donor and recipient PRS into a single predictive model in the transplant setting. Previous studies have investigated the effect of either donor PRS [11] or recipient PRS [13, 15], but none have combined the two in a single predictive model. While the impact of PRS on transplant outcome is relatively modest (accounting for 1% of the variation in recipient post-transplant eGFR), these results align with a growing body of literature demonstrating the utility of PRS in predicting kidney disease [27] and kidney transplant outcome [11]. They are also consistent with recent results demonstrating the association of combined donor and recipient genetic factors with transplant outcome [7, 9, 28]. As GWAS continue to grow in size and predictive power, PRS could potentially explain a more substantial proportion of graft function. It is likely that a GWAS focused on kidney failure would result in significantly better PRS for kidney failure rather than just a GWAS for eGFR, as it is quite possible that the variants involved in low eGFR may be quite different from those involved in kidney failure.

This study has replicated the well described significant impact of clinical factors on long-term graft function and survival including donor age, donor cause of death, HLA mismatch, era of transplantation, and donor type. These clinical factors explain approximately 25% of variation in eGFR at 1-year and significantly outweighs the impact of PRSs on transplant function.

This study has several limitations. The participants included were of predominantly European ancestry. The performance of PRSs in non-European ancestry populations is generally lower, though much work is currently being done to address this issue [28]. Data on HLA mismatch and/or eGFR at 5-year was unavailable on 15% and 50% of participants respectively. We were unable to detect a significant effect of PRSs on graft failure, which may be on account of the effect potentially being stronger in immediate graft function rather than long-term survival. Additionally, 16% of these transplants date from before the year 2000. This means that we have a long follow-up time for many of our transplants, but treatment regimens have improved significantly since some of the earlier transplants in the 1980s and 1990s. We accounted for this by controlling for the year of transplant in our analysis. We lack data on several factors which may influence graft function including history of hypertension, history of diabetes, hepatitis C virus status, terminal serum creatinine, and donor height and weight and thus were unable to calculate the kidney donor profile index (KDPI). However, this is not likely to significantly impact our results, as it has been previously shown that while KDPI was predictive of post-transplant eGFR, it does not significantly add to donor age as a predictor of graft failure [29].

Additionally, the focus in this study is on common variation. Large scale donor-recipient exome studies are currently underway which will address the question of the impact of rare variation on graft function. It is possible, and even likely [30], that incorporating such information on rare variation may yield results of larger effect.

In summary, this study demonstrates that the combined effect of donor and recipient PRSs for decreased eGFR has an impact on post-transplant eGFR. Donors and recipients who both have high PRSs result in an average recipient eGFR at 1-year post-transplant that is over 5 mL/min/1.73 m^2^ lower than the average from transplants where both donor and recipient have average polygenic burden. At this point in their development, these PRSs have minimal added benefit over existing clinical risk factors, but we anticipate that as PRSs become increasingly powerful, that they will become an important tool in clinical decision-making. These results may have potential implications for transplant allocation decisions. Any incorporation of PRSs into such decisions would likely first take place in living donor transplants, where potential donors could be genotyped and analysed without the time pressures that exist around deceased donor transplantation. Before this can take place, further studies are required to validate these results and construct a transplant risk prediction tool based on clinical factors and PRSs.

## Data Availability

The data analyzed in this study is subject to the following licenses/restrictions: Privacy concerns prevent these datasets being made publicly available. Requests to access these datasets should be directed to Graham Lord, graham.lord@manchester.ac.uk.

## Abbreviations

DeKAF, Deterioration of kidney allograft function; eGFR, estimated glomerular filtration rate; FRCBS, Finnish Red Cross Blood Service; GEN03, Genomics of kidney transplantation; GWAS, genome-wide association study; HLA, human leukocyte antigen; KDPI, kidney donor profile index; KiT-GENIE, Kidney transplantation - genomic investigation of essential clinical concerns; PRS, Polygenic risk score; SNP, single nucleotide polymorphism; TKV, total kidney volume; TL, Transplant lines; UKIRTC, United Kingdom and Ireland renal transplant consortium; QUB, Queen’s University Belfast.
