# Supplementary material for: Donor and Recipient Polygenic Risk Scores Influence Kidney Transplant Function
Source: Transpl Int. 2025 Mar 4;38:14171. doi: 10.3389/ti.2025.14171 (PMC11913612; doi:10.3389/ti.2025.14171)
Supplement: Supplementary file 1 [file DataSheet1.docx]

Supplementary materials

[Description of cohorts 2](#_gjdgxs)

[Power analysis 4](#_3znysh7)

[Data quality control and processing 5](#_dxtfze66uex5)

[Details of genome-wide association studies used to generate PRS 7](#_ypa70kfm7lwy)

[References 12](#_pzol5f2js5za)

#

# Description of cohorts

We assembled seven different cohorts for this study, all of primarily European ancestry:

Deterioration of Kidney Allograft Function (DeKAF) and GEN03: The Deterioration of Kidney Allograft Function (DeKAF) and GEN03 studies are cohorts of transplant recipients from 7 transplant centres in the US and Canada[^1^](https://www.zotero.org/google-docs/?jBYd21)^,^[^2^](https://www.zotero.org/google-docs/?9gVCbP). They contributed 1,676 and 998 transplant recipients who all had kidney failure, respectively, to this study.

Finnish Red Cross Blood Service (FRCBS): 929 genotyped transplant recipients transplanted in a single centre in Helsinki between 2007 and 2017[^3^](https://www.zotero.org/google-docs/?lCGgFQ).

Kidney Transplantation - Genomic Investigation of Essential Clinical concerns (KiT-GENIE): 1,831 transplant recipients were collected from the French monocentric KiT-GENIE cohort[^4^](https://www.zotero.org/google-docs/?Dh0NhR).

Queen’s University Belfast (QUB): 150 genotyped transplant recipients from across Northern Ireland. These samples were distinct from the samples included in the UKIRTC[^5^](https://www.zotero.org/google-docs/?LfswtX).

Transplant Lines (TL): 1,056 genotyped transplant recipients from the Netherlands. This cohort is a single-centre biobank from the University Medical Centre Groningen including all different types of solid organ transplant recipients and living organ donors[^6^](https://www.zotero.org/google-docs/?ckm0iW). These individuals were imputed using the 1000 Genomes imputation panel.

United Kingdom and Ireland Renal Transplant consortium (UKIRTC): 2,032 genotyped transplant recipients from all kidney transplant centres in the UK and Ireland that took place between 1987 and 2007[^7^](https://www.zotero.org/google-docs/?whuXJo).

Unless otherwise stated, the autosomal genotypes of all kidney donors and recipients were imputed using either the Haplotype Reference Consortium r1.1 2016 on the Sanger imputation server, with phasing using Eaglev2.4. All data was then filtered for standard QC parameters (Supplementary Materials section on data quality control and processing).

# Power analysis

We performed a power analysis using the *pwr.f2.test* function from the package *pwr* in R to determine the smallest sample size required to reliably detect significant effects, assuming that all 14 PRSs (7 donor PRS + 7 recipient PRS) together explained 1% of the variation in outcome of interest. This was combined with a specification of 95% power and a significance level of 0.05 to generate the smallest sample size required. The smallest sample size required to detect such an effect in the outcome of eGFR at 1-year post-transplant is 272 individuals.

# Data quality control and processing

The following criteria were used to determine inclusion in this study:

1. Standard quality control parameters of minor allele frequency of 0.02, missingness 0.05, genotyping rate of 0.05.
2. All participants were unrelated up to and including the level of 3^rd^ degree. This was determined using KING[^8^](https://www.zotero.org/google-docs/?eUbQsn).

We used the PRSice2 software with a p-value threshold of 0.5 and physical distance threshold for clumping of 250 kb and LD threshold of 0.1 to generate the PRSs.

We chose a P-value threshold of 0.5 for several reasons. Firstly it has been shown that PRSice2, the software that we used, does not give wildly varying results depending on p-value threshold used, unlike some other software[^9^](https://www.zotero.org/google-docs/?PNXfKo). It is also important to note that substantially better predictive power of a PRS can typically be achieved by also including variants that do not reach genome-wide statistical significance[^10^](https://www.zotero.org/google-docs/?WJIDdg) suggestive that many complex traits are influenced by variants with sub threshold effects. One study calculated PRSs using ten different p-value thresholds between 0.0001 and 1 and found largely relatively similar performances of each of the different thresholds[^11^](https://www.zotero.org/google-docs/?NeNSQW). Based on all of these results, we decided to use a p-value threshold of 0.5. We decided to use this threshold, rather than sacrificing a significant amount of our data to do this hyperparamater selection that likely ultimately would not have had a significant effect on our outcomes.

It should also be noted that in some unpublished work that we have done, we have calculated an eGFR PRS using lassosum, and found a correlation over over 80% with the scores calculated using PRSice. This is consistent with previously published results, showing a correlation coefficient of 0.85[^12^](https://www.zotero.org/google-docs/?mmhUF5).

# Details of genome-wide association studies used to generate PRS

Supplementary Table S1: Details of genome-wide association studies used to generate PRS. Sample sizes and reference study for each trait for which a PRS was generated. The number of genome-wide significant loci found in each study is also given. In our analysis, signs were flipped in the total kidney volume and eGFR PRSs in order that a higher PRS would be associated with a negative outcome (i.e. decreased eGFR and decreased total kidney volume).

| Trait | Study | Discovery sample size | Ancestry | SNP based heritability (%) | Effect size | Loci |
| --- | --- | --- | --- | --- | --- | --- |
| Clinical Microalbuminuria (UACR > 30 mg/g) | Teumer et al. 2019[^13^](https://www.zotero.org/google-docs/?d01P9v) | 564,257 | European | 4.3 | OR of 1.69 for quartile 4 vs 1, *P* = 3×10^−191^ | 68 |
| eGFR | Wuttke et al. 2019[^14^](https://www.zotero.org/google-docs/?wrXiLd) | 567,460 | European | 7.1 | OR of chronic renal failure per 10% lower GRS 2.13, *P* = 8.1×10^-38^ | 264 |
| Rapid eGFR decline of > 5 mL/min per 1.73 m^2^/year | Gorski et al. 2020[^15^](https://www.zotero.org/google-docs/?08TMrU) | 19,901 cases, 175,244 controls | All | NA | NA | 7 |
| Total kidney Volume | Liu et al. 2021[^16^](https://www.zotero.org/google-docs/?W8oe4m) | 32,860 | White British | 3.1 | NA | 9 |
| Hypertension | Bi et al. 2020[^17^](https://www.zotero.org/google-docs/?dBrpvI) | 76,566 cases, 206,305 controls | White British | NA | NA | 204 |
| Intracranial aneurysm | Bakker et al. 2020[^18^](https://www.zotero.org/google-docs/?NdDO9C) | 7,495 cases, 71,934 controls | European | 21.6 | NA | 17 |
| Stroke | Malik et al. 2018[^19^](https://www.zotero.org/google-docs/?kGcYd1) | 67,162 cases, 454,450 controls | Mixed | NA | NA | 32 |

UKB: UK Biobank, CKDGen: CKD genetic consortium, MVP: Million Veterans Program.

# Adjusted results by study site

Effect of donor PRSs on eGFR at 1 year


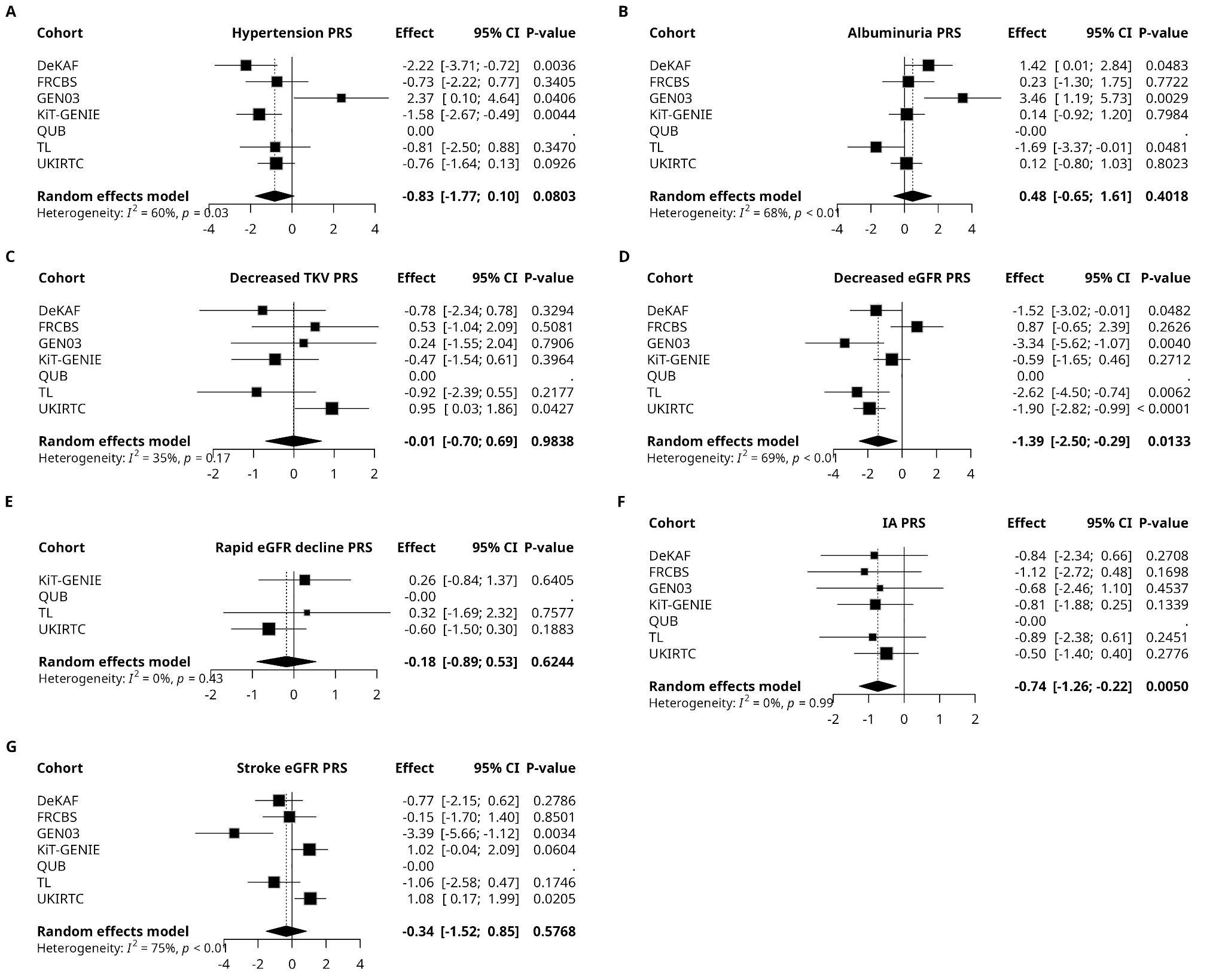


Effect of donor PRSs on eGFR at 5-years


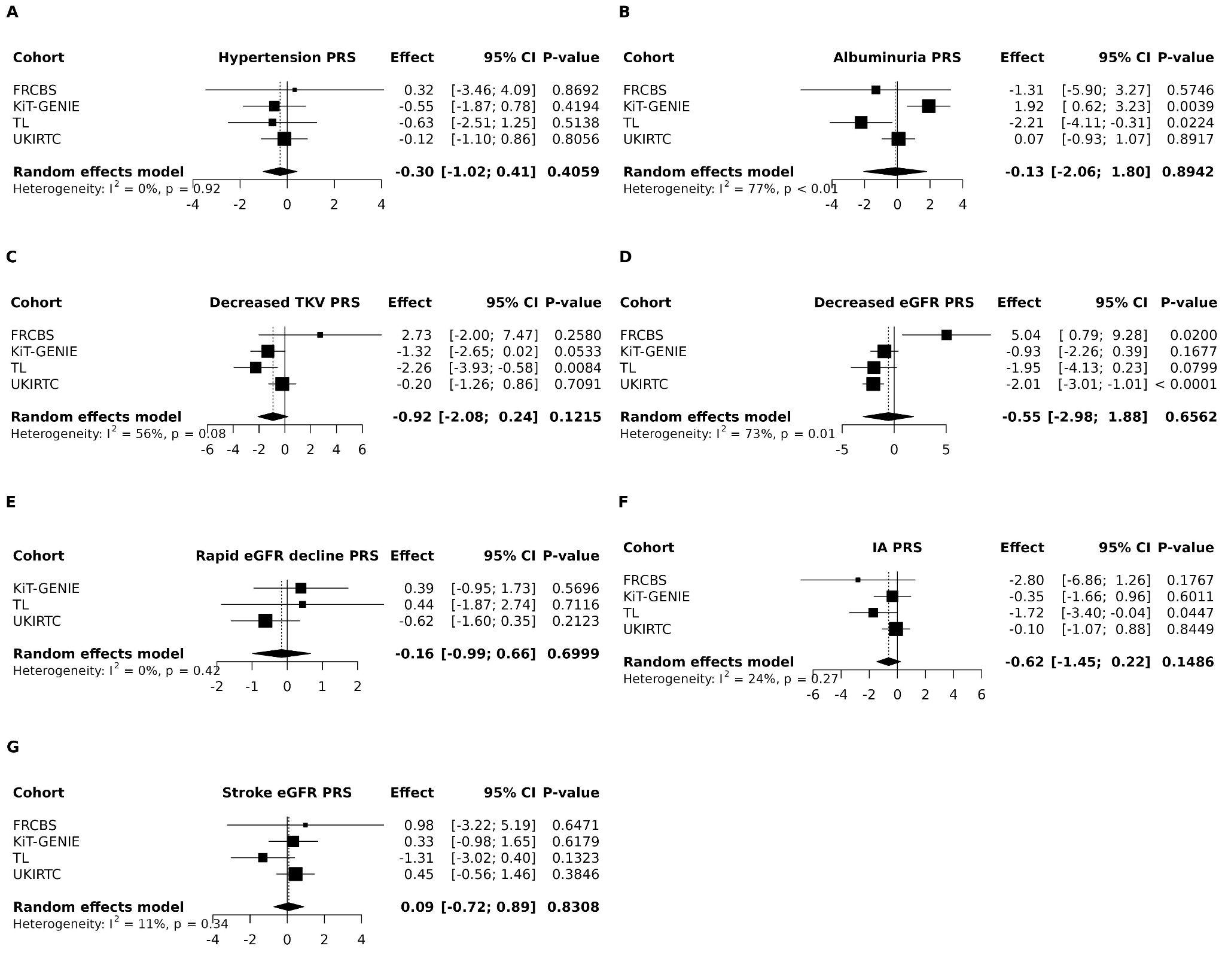


Effect of donor PRSs on graft survival


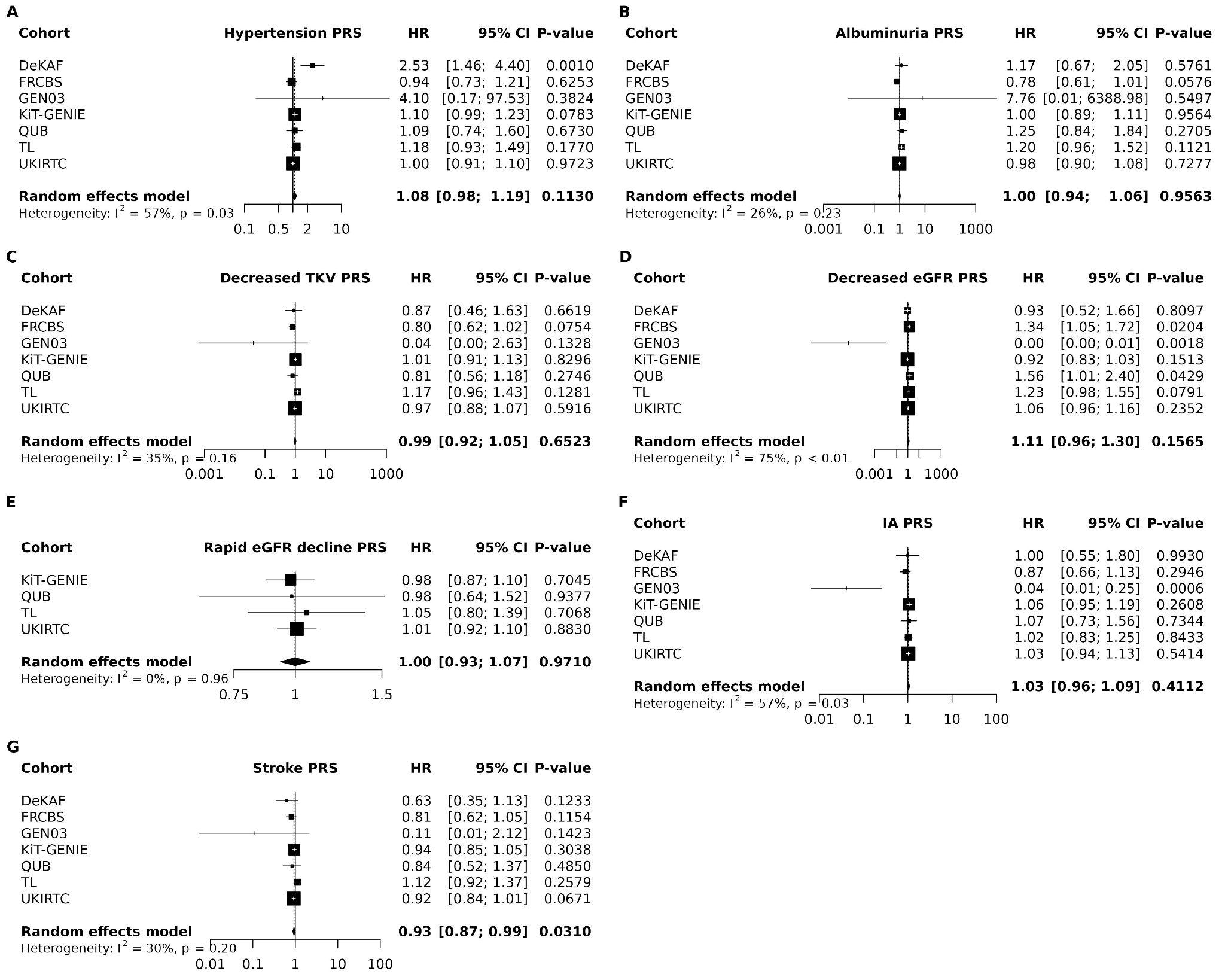


Effect of recipient PRSs on eGFR at 1 year


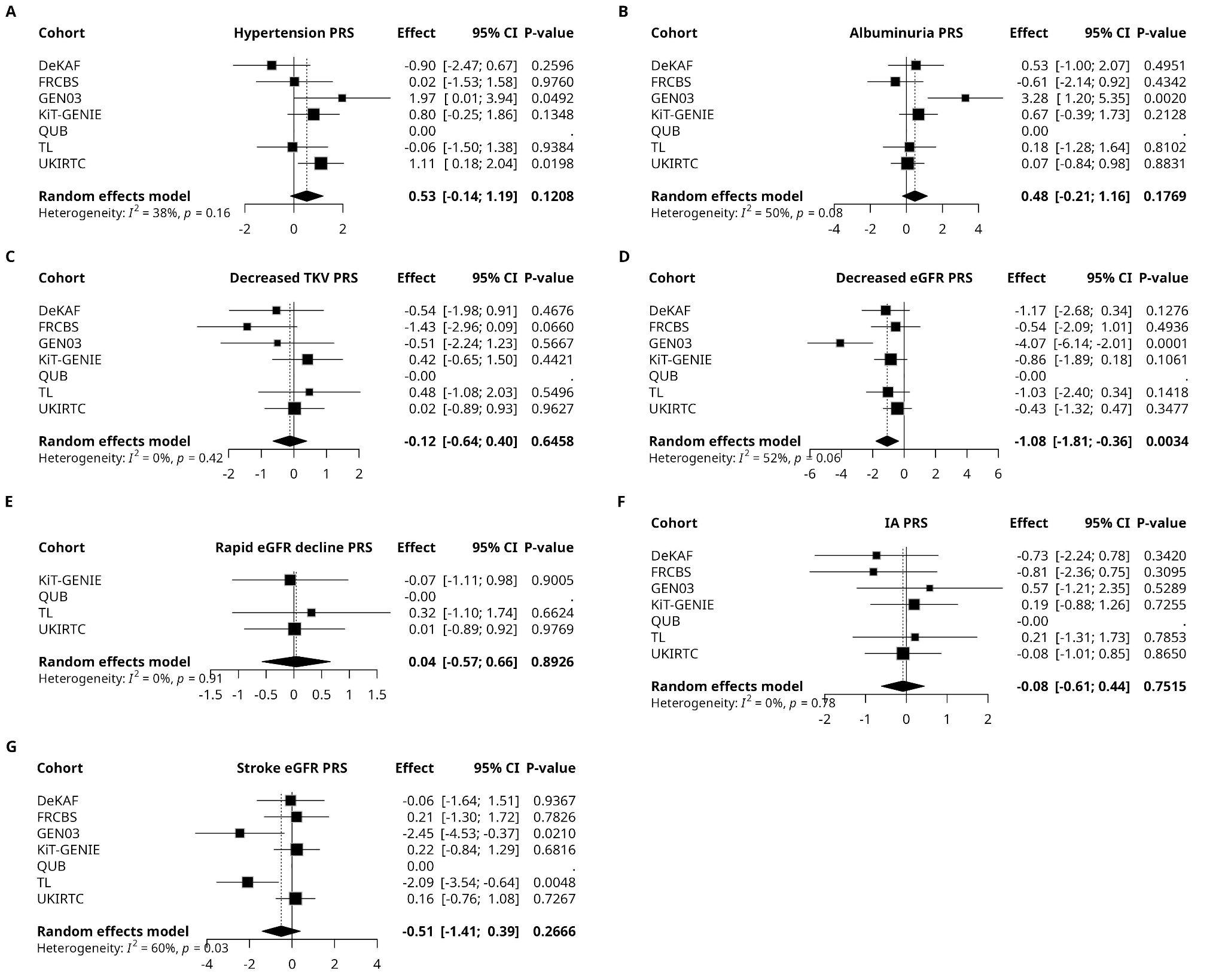


Effect of recipient PRSs on eGFR at 5-years


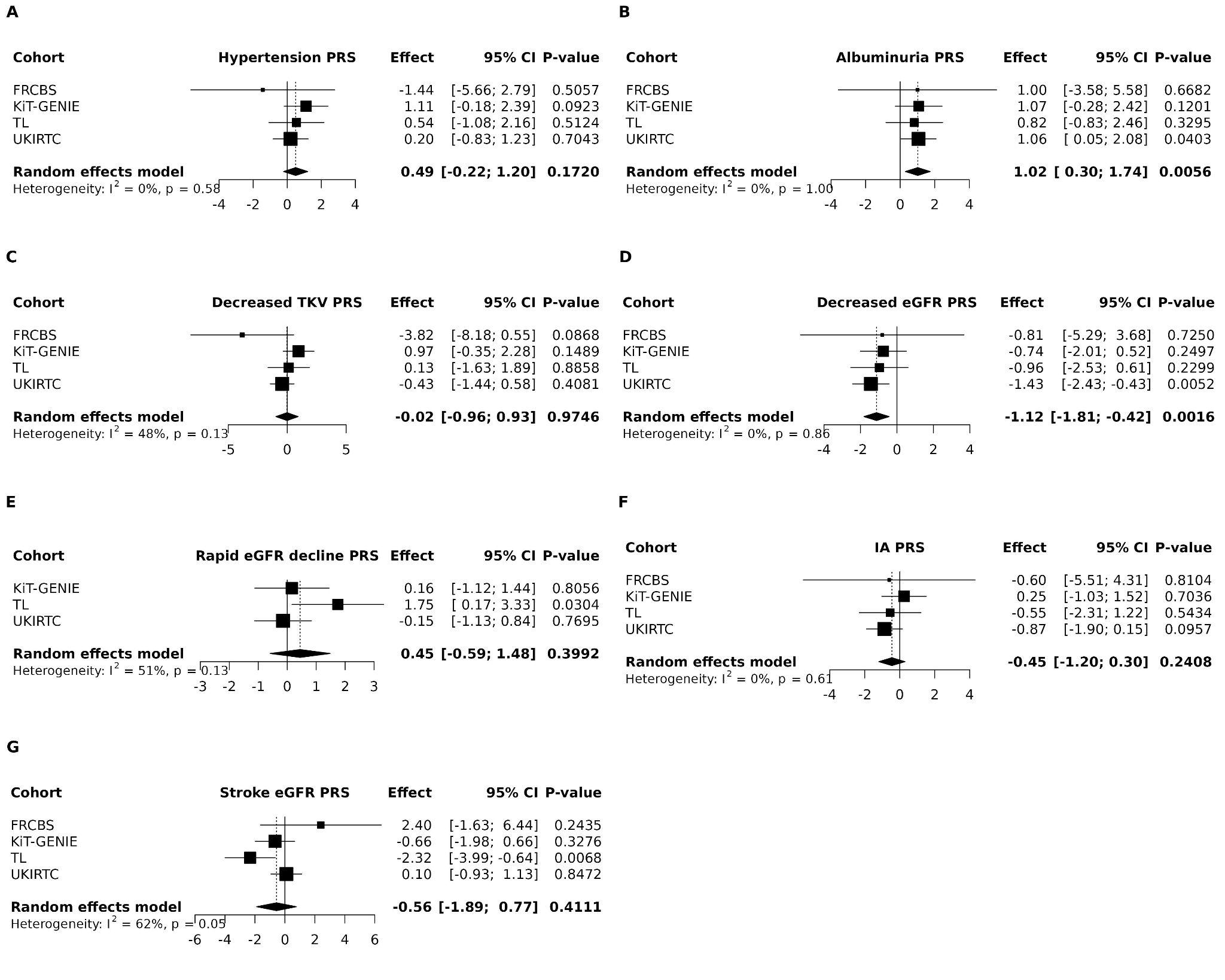


Effect of recipient PRSs on graft survival


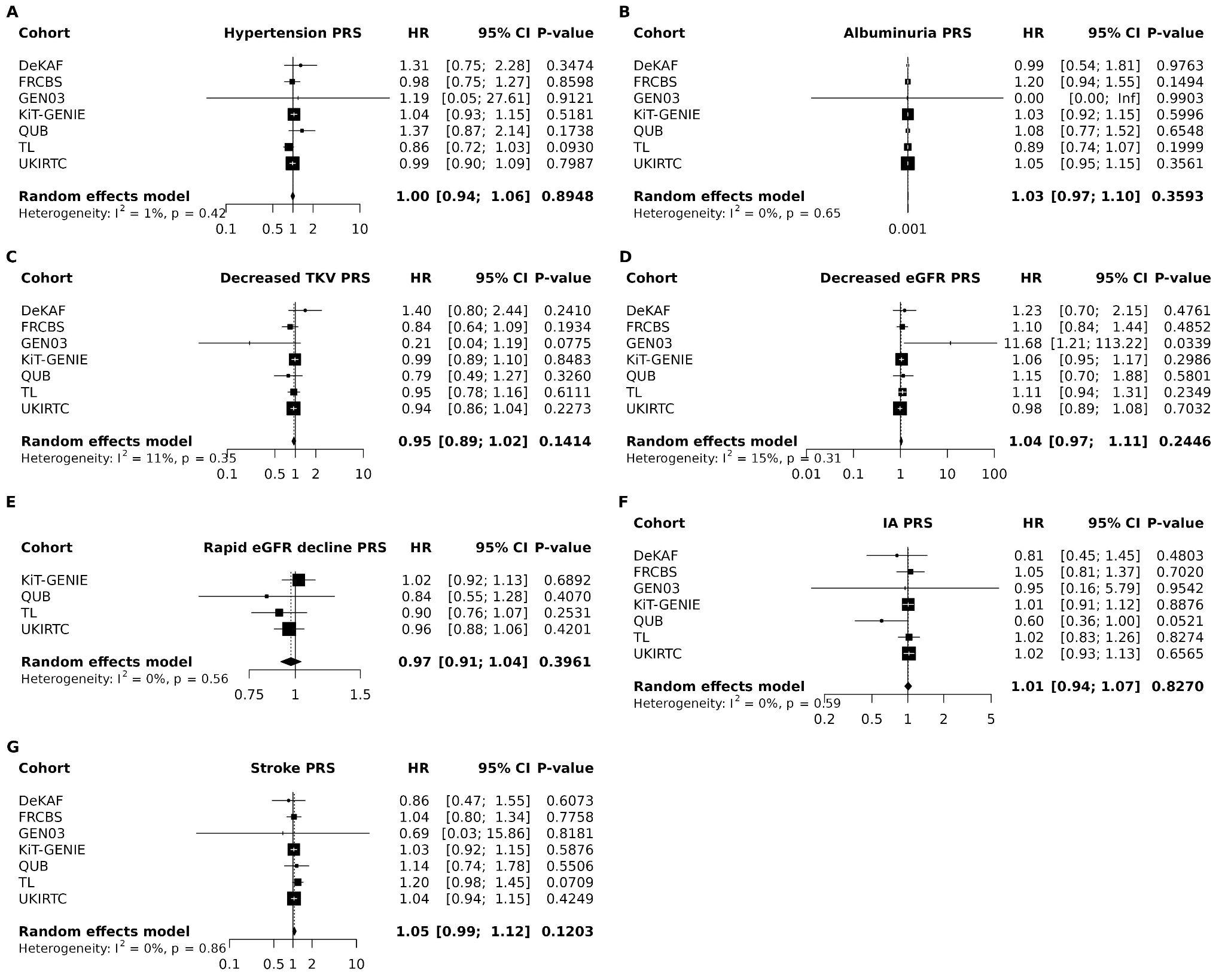


# References

[1. Matas AJ, Fieberg A, Mannon RB, et al. Long-term follow-up of the DeKAF cross-sectional cohort study. *American Journal of Transplantation*. 2019;19(5):1432-1443. doi:10.1111/ajt.15204](https://www.zotero.org/google-docs/?daQ4ZQ)

[2. Mohamed ME, Schladt DP, Guan W, et al. Tacrolimus Troughs and Genetic Determinants of Metabolism in Kidney Transplant Recipients: A comparison of four ancestry groups. *American Journal of Transplantation*. 2019;19(10):2795-2804. doi:10.1111/ajt.15385](https://www.zotero.org/google-docs/?daQ4ZQ)

[3. Markkinen S, Helanterä I, Lauronen J, Lempinen M, Partanen J, Hyvärinen K. Mismatches in Gene Deletions and Kidney-related Proteins as Candidates for Histocompatibility Factors in Kidney Transplantation. *Kidney International Reports*. 2022;7(11):2484-2494. doi:10.1016/j.ekir.2022.08.032](https://www.zotero.org/google-docs/?daQ4ZQ)

[4. Garrigue V, Szwarc I, Giral M, et al. Influence of anemia on patient and graft survival after renal transplantation: Results from the French DIVAT Cohort. *Transplantation*. 2014;97(2):168-175. doi:10.1097/TP.0b013e3182a94a4d](https://www.zotero.org/google-docs/?daQ4ZQ)

[5. Steers NJ, Li Y, Drace Z, et al. Genomic Mismatch at LIMS1 Locus and Kidney Allograft Rejection . *New England Journal of Medicine*. 2019;380(20):1918-1928. doi:10.1056/nejmoa1803731](https://www.zotero.org/google-docs/?daQ4ZQ)

[6. Eisenga MF, Gomes-Neto AW, Van Londen M, et al. Rationale and design of TransplantLines: A prospective cohort study and biobank of solid organ transplant recipients. *BMJ Open*. 2018;8(12):1-13. doi:10.1136/bmjopen-2018-024502](https://www.zotero.org/google-docs/?daQ4ZQ)

[7. Hernandez-Fuentes MP, Franklin C, Rebollo-Mesa I, et al. Long- and short-term outcomes in renal allografts with deceased donors: A large recipient and donor genome-wide association study. *American Journal of Transplantation*. 2018;18(6):1370-1379. doi:10.1111/ajt.14594](https://www.zotero.org/google-docs/?daQ4ZQ)

[8. Manichaikul A, Mychaleckyj JC, Rich SS, Daly K, Sale M, Chen WM. Robust relationship inference in genome-wide association studies. *Bioinformatics*. 2010;26(22):2867-2873. doi:10.1093/bioinformatics/btq559](https://www.zotero.org/google-docs/?daQ4ZQ)

[9. Elgart M, Lyons G, Romero-Brufau S, et al. Non-linear machine learning models incorporating SNPs and PRS improve polygenic prediction in diverse human populations. *Commun Biol*. 2022;5(1):856. doi:10.1038/s42003-022-03812-z](https://www.zotero.org/google-docs/?daQ4ZQ)

[10. The International Schizophrenia Consortium. Common polygenic variation contributes to risk of schizophrenia and bipolar disorder. *Nature*. 2009;460(7256):748-752. doi:10.1038/nature08185](https://www.zotero.org/google-docs/?daQ4ZQ)

[11. Holliday EG, Traylor M, Malik R, et al. Polygenic Overlap Between Kidney Function and Large Artery Atherosclerotic Stroke. *Stroke*. 2014;45(12):3508-3513. doi:10.1161/STROKEAHA.114.006609](https://www.zotero.org/google-docs/?daQ4ZQ)

[12. Yu Z, Jin J, Tin A, et al. Polygenic Risk Scores for Kidney Function and Their Associations with Circulating Proteome, and Incident Kidney Diseases. *JASN*. 2021;32(12):3161-3173. doi:10.1681/ASN.2020111599](https://www.zotero.org/google-docs/?daQ4ZQ)

[13. Teumer A, Li Y, Ghasemi S, et al. Genome-wide association meta-analyses and fine-mapping elucidate pathways influencing albuminuria. *Nature Communications*. 2019;10(1). doi:10.1038/s41467-019-11576-0](https://www.zotero.org/google-docs/?daQ4ZQ)

[14. Wuttke M, Li Y, Li M, et al. A catalog of genetic loci associated with kidney function from analyses of a million individuals. *Nature Genetics*. 2019;51(6):957-972. doi:10.1038/s41588-019-0407-x](https://www.zotero.org/google-docs/?daQ4ZQ)

[15. Gorski M, Jung B, Li Y, et al. Meta-analysis uncovers genome-wide significant variants for rapid kidney function decline. *Kidney International*. 2021;99(4):926-939. doi:10.1016/j.kint.2020.09.030](https://www.zotero.org/google-docs/?daQ4ZQ)

[16. Liu Y, Basty N, Whitcher B, et al. Genetic architecture of 11 organ traits derived from abdominal MRI using deep learning. *eLife*. 2021;10:1-30. doi:10.7554/eLife.65554](https://www.zotero.org/google-docs/?daQ4ZQ)

[17. Bi W, Fritsche LG, Mukherjee B, Kim S, Lee S. A Fast and Accurate Method for Genome-Wide Time-to-Event Data Analysis and Its Application to UK Biobank. *Journal of Cleaner Production*. 2020;107(2):222-233. doi:10.1016/j.ajhg.2020.06.003](https://www.zotero.org/google-docs/?daQ4ZQ)

[18. Bakker MK, van der Spek RAA, van Rheenen W, et al. Genome-wide association study of intracranial aneurysms identifies 17 risk loci and genetic overlap with clinical risk factors. *Nature Genetics*. 2020;52(12):1303-1313. doi:10.1038/s41588-020-00725-7](https://www.zotero.org/google-docs/?daQ4ZQ)

[19. Malik R, Chauhan G, Dichgans M. Multiancestry genome-wide association study of 520,000 subjects identifies 32 loci associated with stroke and stroke subtypes. *Nature Genetics*. 2018;50(12):524-537. doi:10.1038/s41588-018-0058-3](https://www.zotero.org/google-docs/?daQ4ZQ)
